# Impact of catheter-directed thrombolysis access approach on entire-limb deep vein thrombosis: a focus on inflow patency

**DOI:** 10.3389/fcvm.2025.1663587

**Published:** 2025-10-31

**Authors:** Cheng Qian, Wei-Qing Jiang, Kang Guo, Tao Wang, Wen-Sheng Lou, Ying-Hao Li, Jian-Ping Gu, Guo-Ping Chen

**Affiliations:** ^1^ Department of Interventional Radiology, Nanjing First Hospital, Nanjing Medical University, Nanjing, China; ^2^ Department of Anesthesiology, Nanjing First Hospital, Nanjing Medical University, Nanjing, China; ^3^ Department of Radiology, Yixing Traditional Chinese Medicine Hospital, Wuxi, China

**Keywords:** catheter-directed thrombolysis, entire-limb deep venous thrombosis, post-thrombotic syndrome, inflow patency, propensity-score matching

## Abstract

**Objective:**

This retrospective study aimed to evaluate the influence of inflow (femoropopliteal) patency on the efficacy of catheter-directed thrombolysis (CDT) in treating entire-limb deep venous thrombosis (DVT).

**Methods:**

From January 2018 to December 2022, 121 individuals diagnosed with acute entire-limb DVT were treated with CDT. CDT was performed through the ipsilateral popliteal vein (AGA) or the contralateral common femoral vein (RGA). Baseline characteristics and segmental patency were compared between the two access approaches groups. The severity of post-thrombotic syndrome (PTS) was compared between different inflow patency groups. Propensity-score matching (PSM) was used to balance confounding factors. Potential risk factors for PTS were analyzed using univariate and multivariate regression analysis.

**Results:**

Thirty-four patients received the AGA approach, while 87 individuals were treated with the RGA approach. The AGA group had superior patency compared to the RGA group in both the popliteal and femoral veins (*P* < 0.0001). “Good” inflow (great and fair patency) was associated with a lower PTS incidence and severity compared to “bad” inflow (poor patency) (*P* < 0.0001). Most patients with “bad” inflow (94.1%) received the retrograde approach. The PSM analysis yielded 97 well-matched pairs (59 patients in the “good” inflow group, and 38 in the “bad” inflow group). The access approach did not significantly affect PTS rate. Multivariate analysis identified “bad” inflow patency as a predictor of PTS (OR: 3.41, 95% CI: 1.94–6, *P* < 0.0001), further treatment showed a protective effect (OR: 0.17, 95% CI: 0.1–0.3, *P* < 0.0001).

**Conclusion:**

“Good” inflow patency decreased the incidence and degree of PTS among patients with entire-limb DVT.

## Introduction

Acute lower limb deep vein thrombosis (DVT) is a common vascular disease, affecting approximately 1 in 1,000 individuals (1). Incomplete thrombus dissolution may lead to post-thrombotic syndrome (PTS), especially in cases of acute entire-limb (from calf to iliac vein) DVT (2, 3). The European Society for Vascular Surgery guidelines recommend early thrombus removal strategies (4), as they can improve the quality of life and reduce the severity of PTS (5–9). Catheter-directed thrombolysis (CDT) is an effective method for early thrombus removal. A cost-effectiveness analysis has shown that CDT is more cost-effective than anticoagulation alone (9). Different CDT access techniques result in varying treatment outcomes (10). However, the underlying mechanisms remain unclear. The inflow status is a crucial determinant of iliac vein stent patency. For patients undergoing iliac vein stent implantation, those with “good” inflow have better patency than those with “fair/poor” inflow (11–14). Some studies have suggested that the inflow status affects the efficacy of CDT. Inflow (femoropopliteal) thrombosis blocks upstream blood flow, hindering the efficient delivery of thrombolytic drugs and increasing the risk of occlusive thrombus formation within the iliac veins (15). However, Jeyabalan et al. reported that inflow thrombosis does not affect the efficacy of CDT (16). The results may have been biased by factors such as 45.4% of patients having symptoms for more than 14 days and 72% of participants undergoing percutaneous mechanical thrombectomy (PMT) treatment. Therefore, the role of inflow patency in CDT treatment requires further investigation.

This retrospective comparative study was conducted to assess the impact of inflow (femoropopliteal) patency on the clinical efficacy of CDT for the treatment of acute entire-limb DVT.

## Materials and methods

### Study design and study population

This retrospective study was approved by the institutional review board. The clinical data of patients with entire-limb DVT receiving CDT treatment in our department from January 2018 to December 2022 were collected.

### Inclusion and exclusion criteria

The inclusion criteria were as follows: (1) unilateral entire-limb DVT; (2) first episode of DVT; (3) a symptomatic period less than 14 days; (4) receiving CDT treatment; (5) a minimum follow-up period of 12 months. The exclusion criteria were: (1) symptom duration longer than 14 days or history of DVT; (2) bilateral DVT; (3) combined PMT; (4) CDT with both antegrade and retrograde accesses. Finally, 121 patients were included in the study.

### Procedures

Once diagnosed, patients were administered low-molecular-weight heparin at a dosage of 100 IU/kg per 12 h. The interventional procedures were conducted by two radiologists, each with 2–5 years of practice experience. It was a standard practice to implant a retrievable inferior vena cava filter (IVCF) before starting CDT (17).

For the antegrade approach (AGA), the ipsilateral popliteal vein was selected. After IVCF placement, the patient was placed in the prone position. Then, under local anesthesia and venographic guidance, a 5 French sheath was inserted. Subsequently, a multiple-holes infusion catheter (4 French Uni*Fuse Infusion Catheter, AngioDynamics, USA, or 5 French Infusion Catheter, COOK, USA) was inserted, with its tip placed into the inferior vena cava. The retrograde approach (RGA) was performed through the contralateral common femoral vein. An Omini, Cobra, or Simon catheter was used to explore the entrance of the ipsilateral common iliac vein, and then the infusion catheter was introduced to the distal popliteal vein.

The selection of the access approach was mainly based on the following clinical factors:

(1) Vascular anatomical conditions: The anatomical structure of the patient's blood vessels was evaluated by preoperative imaging examinations. If there were anatomical abnormalities in the ipsilateral popliteal vein (such as stenosis, tortuosity, or variation) that made it difficult to puncture and insert the catheter, the RGA approach was selected.
(2) Physician's clinical experience and judgment: The interventional radiologists performing the procedure comprehensively considered the above factors combined with their own clinical experience. For patients with complex conditions, a joint discussion was conducted by the team of interventional radiologists to determine the most appropriate access approach to ensure the safety and effectiveness of the operation.

Urokinase (Lizhu Pharmaceutical Factory, China) was used as the thrombolysis agent. The dosage and usage were based on the Chinese expert consensus (17) and were described in a previous study (10). Thrombus burden was assessed daily by venography. Fibrinogen levels, hemoglobin levels and platelet counts were monitored daily. If the fibrinogen level dropped below 1.5 g/L, the dosage was reduced; if it dropped below 1 g/L, thrombolysis was discontinued. The thrombolysis procedure, which lasted one week, was terminated when complete thrombolysis was achieved or when two consecutive assessments showed no change. For cases of iliac vein compression or occlusion, further treatment (balloon dilatation or balloon dilatation+ stent implantation) was performed. If the residual stenosis was greater than 50% after balloon dilatation, a self-expandable stent (12–16 mm, Wallstent, Boston Scientific, USA) was implanted. After the thrombolysis process, the patients continued to receive anticoagulation therapy with warfarin or rivaroxaban.

### Evaluation

Treatment efficacy was evaluated using a modified SVS scoring system (10). Scores were defined as follows: 0 for a patent vein without thrombus; 1 for partial occlusion with thrombus present; 2 for complete occlusion. The clot burden reduction rate was calculated as (pre-CDT limb scores—post-CDT scores)/pre-CDT score × 100%. Thrombus burden reduction was categorized into grades (10): Grade I for a reduction of less than 50%; Grade II for partial thrombolysis, corresponding to a 50%–95% reduction; Grade III for a 95%–100% reduction or complete lysis. Inflow patency was classified as follows (11): Inflow was classified as “poor” when the inflow vessels small caliber inflow vessels with many collateral vessels or even the trunk was undetected. “Great” indicated no post-thrombotic changes in adequate caliber inflow vessels. “Fair” indicated mild post-thrombotic changes. Specifically, the caliber of the blood vessel was reduced but the blood flow was still basically smooth, and there was no obvious blood flow stasis. “Great” and “fair” patency inflows were regarded as “good” inflow, while “poor” patency inflow was categorized as “bad” inflow. Venogram image interpretation was performed by three radiologists, each with over 5 years of experience, who were blinded to the intervention.

### Follow-up

All patients were evaluated at 1, 3, 6, and 12 months post-lysis, with annual follow-up visits thereafter. Endpoint events included patient mortality and loss to follow-up at the last recorded visit. PTS was assessed using the Villalta scale (18).

### Statistical analysis

All continuous variables were expressed as means and standard deviations (SDs), while categorical variables were presented as frequencies or percentages. Categorical variables were compared using the Chi-square test, and continuous variables were compared using Student's *t*-test. Statistically significant differences were defined at *P* < 0.05 (two-tailed). Kaplan–Meier analysis was used to calculate the free-PTS time, the log-rank test was used to assess the association between relevant variables and free-PTS. The Cox's hazard multivariate regression model was employed to explore potential independent prognostic factors. Propensity-score matched (PSM) analysis, adjusted for “gender” and “further treatment”, was performed to balance the baseline characteristics between the two inflow groups. The two groups were matched at a 1:2 ratio using the nearest-neighbor method with a caliper width of 0.1. All statistical analyses were conducted using SPSS software (version 17). Univariate and multivariate analyses of PTS incidence after PSM were performed using R (4.3.1).

## Results

### Baseline characteristics

The demographics and risk factors were summarized in Table 1. The mean age of the patients was 56.1 ± 12.5 years, 38.8% (47/121) were male. Seventy-four cases (61.2%) involved the left limb. The leading risk factor was May-Thurner syndrome, present in 59.5% (72/121).

**Table 1:** The baseline characteristics of the patients with entire-limb deep venous thrombosis.

Characteristics	Total (*N* = 121)
Age, years	56.08 (12.448)
Gender	
Male	47 (38.8)
Female	74 (61.2)
Onset time, days	6.293 (3.9768)
Affected limb	
Left	74 (61.2)
Right	47 (38.8)
Risk factors	
Cancer	11 (9.1)
Recent major surgery	20 (16.5)
Immobilization	18 (14.9)
May-Thurner syndrome	72 (59.5)
hypercoagulable states^a^	12 (9.9)

Data presented as mean (SD) or No. (%). a, other hypercoagulable state, such as nephrotic syndrome, anti-phospholipid syndrome, and so on.

### Comparison of inflow patency rates between the two access groups

Thirty-four patients underwent CDT via the AGA approach, while 87 received the RGA method (Table 2). The AGA group had significantly better inflow patency than the RGA group. For the popliteal vein, the AGA group had a much higher rate of Grade III patency (76.5%) compared to the RGA group (4.6%). For the femoral vein, the AGA group also showed superior patency rates (Grade III: 67.6%, Grade II: 23.5%, Grade I: 8.8%) compared to the RGA group (Grade III: 1.1%, Grade II: 44.8%, Grade I: 54.0%) (*P* < 0.0001). Overall, the AGA group showed a higher rate of great inflow patency (67.6%) and lower rates of fair (23.5%) and poor (8.8%) inflow patency compared to the RGA group (1.1% in great, 43.7% in fair, and 55.2% in poor) (*P* < 0.0001).

**Table 2:** Comparison of inflow patency of antegrade and retrograde access approach.

Section	AGA Group (*N* = 34)	RGA Group (*N* = 87)	*P* value
Popliteal vein			0.0001
Grade III	26 (76.5)	4 (4.6)	
Grade II	7 (20.6)	61 (70.1)	
Grade I	1 (2.9)	22 (25.3)	
Femoral vein			0.0001
Grade III	23 (67.6)	1 (1.1)	
Grade II	8 (23.5)	39 (44.8)	
Grade I	3 (8.8)	47 (54.0)	
Inflow patency			0.0001
Great	23 (67.6)	1 (1.1)	
Fair	8 (23.5)	38 (43.7)	
Poor	3 (8.8)	48 (55.2)	

Data presented as mean (SD). AGA group, antegrade approach group; RGA group, retrograde approach group.

### Comparison of baseline characteristics in different inflow patency groups

Table 3 showed the differences in variuos characteristics between the “good” and “bad” inflow groups. Most patients with “bad” inflow (94.1%) received the retrograde approach, while only three patients (5.9%) with “bad” inflow received the antegrade approach. The “good” inflow group had a lower incidence and less severity PTS compared to the “bad” inflow group (*P* < 0.0001). Since there were differences in gender and further treatment between the two groups, PSM analysis was performed to balance these two potential confounding factors. As a result, 97 well-matched pairs were obtained, with 59 patients in the “good” inflow group and 38 in the “bad” inflow group. After PSM, the “good” inflow group still had a lower incidence and less severe PTS compared to the “bad” inflow group (*P* < 0.0001).

**Table 3:** Comparison of baseline characteristics of unmatched and matched DVT patients with different inflow patency.

Characteristics	Unmatched patients			Matched patients		
	Patients with “good” inflow (*N* = 70)	Patients with “bad” inflow (*N* = 51)	*P* value	Patients with “good” inflow (*N* = 59)	Patients with “bad” inflow” (*N* = 38)	*P* value
Age (years)	55.9 (12.324)	56.33 (12.735)	0.851	54.6 (12.549)	56.32 (12.747)	0.522
Gender			0.028			0.965
Male	33 (47.1)	14 (27.5)		22 (37.3)	14 (36.8)	
Female	37 (52.9)	37 (72.5)		37 (62.7)	24 (63.2)	
Onset time days)	6.536 (3.968)	5.961 (4.005)	0.435	6.432 (3.8846)	6.395 (4.1365)	0.964
Affected limb			0.408			0.465
Left	45 (64.3)	29 (56.9)		37 (62.7)	21 (55.3)	
Right	25 (35.7)	22 (43.1)		22 (37.3)	17 (44.7)	
Access approach			0.0001			0.0001
Antegrade	31 (44.3)	3 (5.9)		27 (45.8)	3 (7.9)	
Retrograde	39 (55.7)	48 (94.1)		32 (54.2)	35 (92.1)	
Access establishment time (mins)	19.04 (12.914)	21.88 (8.211)	0.17	18.61 (12.968)	23.74 (8.5)	0.034
Urokinase (U)	369.93 (50.105)	371.67 (52.836)	0.854	366.02 (49.938)	370.53 (53.433)	0.674
Further treatment	58 (82.9)	26 (51.0)	0.0001	47 (79.7)	26 (68.4)	0.21
PTS			0.0001			0.0001
No	48 (68.3)	8 (15.7)		38 (64.4)	8 (21.1)	
PTS severity			0.0001			0.0001
Mild	17 (24.3)	16 (31.4)		16 (27.1)	13 (34.2)	
Moderate	2 (2.9)	22 (43.1)		2 (3.4)	13 (34.2)	
Severe	3 (4.3)	5 (9.8)		3 (5.1)	4 (10.5)	

AGA group, antegrade approach group; RGA group, retrograde approach group. Data presented as mean (SD) or number (%).

### Impact of access approach and inflow patency on PTS

**Figure 1:**
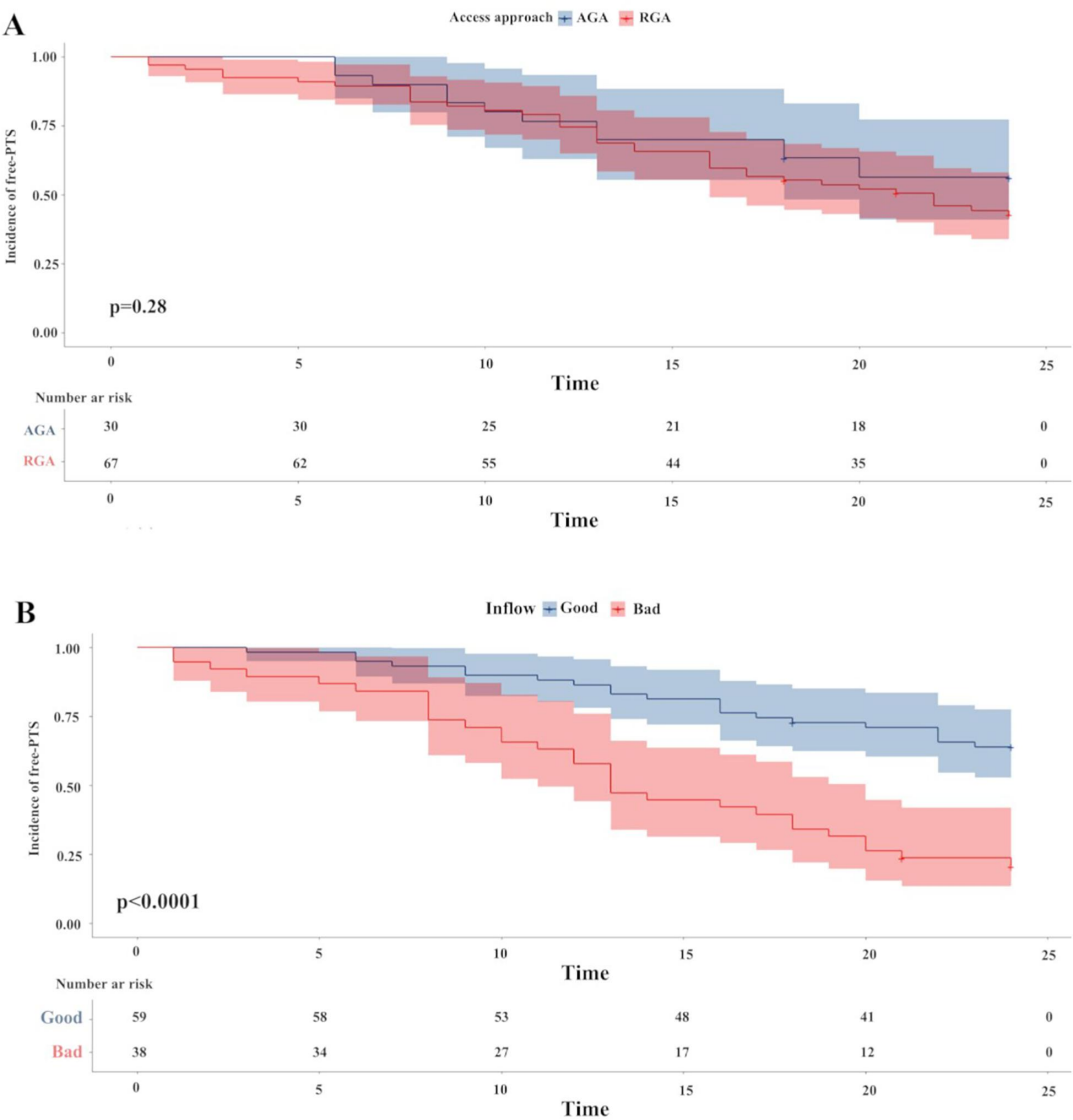
The Kaplan–Meier survival plots of incidence of free-PTS. There was no difference between the two access approach groups (*P* = 0.28) (Figure 1A). A significantly lower 2-year free-PTS was observed among patients in the “good” inflow patency after PSM. *P*-value was calculated by log-rank test and indicated in the plot. PTS, post-thrombotic syndrome; PSM, propensity score matching.

After PSM, the impact of the access approach and inflow patency on the incidence and severity of PTS were assessed (Table 4). There was no significant difference in the incidence, severity and two00 year cumulative incidence of PTS between the two access groups (Figure 1A). However, the “good” inflow patency group had a lower incidence of PTS (35.6%) than the “bad” inflow patency group (78.9%) (*P* < 0.0001). Moreover, the severity of PTS in the “good” inflow patency group was milder than that in the “bad” inflow patency group. A significant difference was observed in the two- year cumulative incidence of PTS between the two groups (*P* < 0.0001) (Figure 1B).

**Table 4:** Comparison of the incidence of post-thrombotic syndrome (PTS) concerning different access approaches and inflow patency after propensity score matching.

Characteristics	Access approaches			Inflow patency		
	AGA group (*N* = 70)	RGA group (*N* = 51)	*P* value	“Good” inflow (*N* = 59)	“Bad” inflow (*N* = 38)	*P* value
PTS			0.222			0.0001
No	17 (56.7)	29 (43.3)		38 (64.4)	8 (21.1)	
PTS severity			0.617			0.0001
Mild	8 (26.7)	21 (31.3)		16 (27.1)	13 (34.2)	
Moderate	3 (10.0)	12 (17.9)		2 (3.4)	13 (34.2)	
Severe	2 (6.7)	5 (7.5)		3 (5.1)	4 (10.5)	

AGA group, antegrade approach group; RGA group, retrograde approach group. Data presented as mean (SD).

### Possible risk factors for PTS

After PSM, a total of 51 patients (52.6%) developed PTS during the two-year follow-up. Univariate analysis showed that further treatment had a protective effect [odd ratio (OR) 0.16, 95% CI: 0.09–0.29, *P* < 0.0001]. Further analysis revealed that balloon dilatation alone (OR: 0.21, 95% CI: 0.03–1.53, *P* = 0.123) and balloon dilatation + stent implantation (OR: 0.16, 95% CI: 0.09–0.29, *P* < 0.0001) were associated with a reduced risk of PTS. In contrast, “bad” inflow patency was identified as a risk factor for PTS occurrence (OR: 3.55, 95% CI: 2.02–6.25, *P* < 0.0001). Additionally, poor inflow (OR: 6.6, 95% CI: 2.31–18.83, *P* = 0.0001) was associated with an increased PTS risk (Table 5).

**Table 5:** Univariate analysis of PTS occurrence after PSM.

Characteristics	OR	95% CI	*P* value
Gender (male)	0.81	0.46–1.42	0.46
Age (>60 year)	0.94	0.54–1.63	0.822
Oneset time (>7 day)	1.19	0.69–2.07	0.53
Side (left)	1.11	0.63–1.96	0.706
Access approach (AGA)	1.41	0.75–2.64	0.289
Further treatment			
No			
Yes	0.16	0.09–0.29	0.0001
Further treatment			
No			
PTA	0.21	0.03–1.53	0.123
Stent	0.16	0.09–0.29	0.0001
Inflow			
“good”			
“bad”	3.55	2.02–6.25	0.0001
Inflow			
Great			
Fair	2.32	0.78–6.9	0.13
Poor	6.6	2.31–18.83	0.0001

Multivariate regression analysis also demonstrated that further treatment had a protective effect (OR: 0.17, 95% CI: 0.1–0.3, *P* < 0.0001), with balloon dilatation alone (OR: 0.17, 95% CI: 0.02–1.3, *P* = 0.088) and balloon dilatation + stent implantation (OR: 0.16, 95% CI: 0.09–0.29, *P* < 0.0001). Conversely, “bad” inflow patency increased the risk of PTS (OR: 3.41, 95% CI: 1.94–6, *P* < 0.0001), and poor patency (OR: 6.88, 95% CI: 2.4–19.72, *P* < 0.0001) (Table 6).

**Table 6:** Multivariate analysis of PTS occurrence after PSM.

Characteristics	OR	95% CI	*P* value	Characteristics	OR	95% CI	*P* value
Further treatment				Further treatment			
No				No			
Yes	0.17	0.1−0.3	0.0001	PTA	0.17	0.02–1.3	0.088
				Stent	0.16	0.09–0.29	0.0001
Inflow				Inflow			
“good”				Great			
				Fair	2.63	0.88–7.88	0.084
“bad”	3.41	1.94–6	0.0001	Poor	6.88	2.4–19.72	0.0001

## Discussion

CDT is recognized as a low-risk and efficacious treatment for DVT (19). Previous research has shown that the access approach affects the effectiveness of CDT (10), but the underlying mechanisms remain unclear. In previous study, both proximal and entire-limb DVT patients were included, the different types of DVT may have influenced the treatment outcome. To clarify this confounding factor, this study focused on acute entire-limb DVT. The results of this study indicate that inflow patency plays an important role in the access approach and has a notable impact on the incidence and severity of PTS. Therefore, achieving optimal inflow patency can reduce the occurrence and severity of PTS in patients with acute entire-limb DVT.

Although studies have shown that PMT has an advantage over simple CDT in completely or largely removing thrombi (7–9), not all patients can receive PMT due to equipment limitations in local hospitals or patient financial constraints. Thus, CDT-based endovascular treatment remains one of the effective treatment options.

This study found that the AGA group had a higher patency rate than the RGA group in the popliteal and femoral veins, suggesting that different access approaches affect inflow patency. There are several possible reasons for this. In the antegrade approach, the thrombolytic agent is initially directed into the occluded femoral-popliteal vein and then flows to upstream vessels. In contrast, in the retrograde approach, due to the high pressure in the distal thrombus segment, most of the thrombolytic agent escapes through the side holes of the infusion catheter into collateral vessels before reaching the downstream vein. This leads to insufficient thrombolytic agents for dissolving the distal thrombus, resulting in a lower patency rate of the inflow pathways in the RGA group compared to the AGA group. However, Ni et al. reported that patients treated with CDT following PMT had a lower incidence of PTS in the RGA group than those in the AGA group (20). This might be because PMT rapidly removes the inflow thrombus, reducing the distal venous pressure and allowing the thrombolytic agent to reach the distal vessel more easily. This further emphasizes the importance of inflow patency.

Inflow patency significantly affects the long-term effectiveness of CDT. Previous research has suggested that one of the fundamental principles of CDT is to maintain continuous upstream blood flow to keep the cleared vein segments open. Other studies have indicated that the initial status of the inflow is a crucial factor in determining subsequent venous hemodynamics in patients with PTS (21–23). Inflow disease has been identified as the most significant predictor of re-intervention in post-thrombotic syndrome, with an odd ratio of 3.57 [95% confidence interval (CI): 1.26–10.13, *P* < 0.017) (13, 24). Ensuring adequate inflow capacity and velocity within iliofemoral stents is essential to minimize the occurrence of re-occlusion or thrombosis (14). The severity of PTS is closely related to the level of inflow patency. This study further demonstrates that the incidence and severity of PTS are significantly higher in the “bad” inflow patency group than in the “good” inflow patency group. Most patients in the “bad” inflow patency group received retrograde approach CDT, which highlights the impact of inflow patency on the occurrence and severity of PTS. Previous research has shown that RGA group has a better thrombolysis effect in iliofemoral DVT compared to entire-limb DVT, indicating the extent of thrombosis affects thrombolytic efficacy (10). The antegrade approach shows consistently good thrombolysis effects in both extents of thrombosis. These findings indirectly confirm the importance of inflow patency.

Although there is difference in the degree of inflow patency between the two CDT access approaches, the incidence and severity of PTS did not differ significantly between the two groups. One possible reason is that the sample size of this study is relatively small, and further validation with a larger case dataset is needed. Additionally, there may be other potential factors influencing the outcomes that were not considered in this study. Comorbidities such as diabetes, hypertension, and heart failure may affect the patient's vascular condition, blood coagulation function, and wound healing ability, thereby influencing the efficacy of CDT and the occurrence of PTS. Some patients may have received other treatments such as hormonotherapy, antineoplastic drugs, oral contraception and so on. These treatments may also influence the results of CDT.

It should be noted that our study has limitations due to its retrospective design. Retrospective studies rely on existing clinical data, making it impossible to fully control all variables that may affect the research results and thus limiting the ability to establish a clear causal relationship between the research factors and outcomes. Therefore, prospective, randomized controlled trials (RCTs) are needed to further confirm our findings. Second, the small sample size may lead to the inability to detect some potential differences between groups or the overestimation/underestimation of the effect size of the research factors. Multicenter, large-sample study will be carried out to reduce the impact of random errors, and more reliably confirm the conclusions of this study. The incidence of PTS was only assessed at the 2-year mark, and long-term outcomes remain undocumented. This study would continue to follow up the patients included in this study, extend the follow-up time to 5 years or more, and regularly assess indicators such as the incidence and severity of PTS, vascular patency, and the occurrence of other complications. Although the access approaches affected the inflow patency and inflow patency was closely related to the outcomes of CDT, the access approaches did not directly influence the incidence of PTS, suggesting that there may be other contributing factors not accounted for in this study.

## Conclusion

“Bad” inflow patency is associated with an increased incidence and severity of PTS. Further treatment post-CDT plays a protective role. These findings contribute to the refinement of treatment strategies for lower limb DVT, highlighting the importance of optimal inflow patency in CDT interventions.

## Data Availability

The raw data supporting the conclusions of this article will be made available by the authors, without undue reservation.
